# Preharvest Control of *Campylobacter* Colonization in Chickens, with a Special Emphasis on Vaccination Strategies

**DOI:** 10.3390/microorganisms13102378

**Published:** 2025-10-15

**Authors:** Chaitanya Gottapu, Lekshmi K. Edison, Gary D. Butcher, Subhashinie Kariyawasam

**Affiliations:** 1 Department of Comparative, Diagnostic, and Population Medicine, College of Veterinary Medicine, University of Florida, Gainesville, FL 32608, USA; cgottapu@ufl.edu (C.G.); edison.le@ufl.edu (L.K.E.); 2 Department of Large Animal Clinical Sciences, College of Veterinary Medicine, University of Florida, Gainesville, FL 32608, USA; butcher@ufl.edu

**Keywords:** *Campylobacter*, foodborne infection, gastroenteritis, gut microbiota, One-Health, poultry, preharvest control strategies, vaccination

## Abstract

*Campylobacter* is a leading cause of human gastroenteritis, with poultry serving as the primary reservoir host. Effective preharvest control strategies are crucial for preventing or reducing *Campylobacter* contamination on meat surfaces. As concerns grow regarding the use of antimicrobials in animal agriculture, the importance of non-antimicrobial preharvest strategies in poultry production has become increasingly significant. This comprehensive review focuses on the biology of *Campylobacter*, its impact on public health, and current and emerging preharvest strategies, with a special emphasis on vaccination. Preharvest strategies are broadly classified into biosecurity measures, gut microbiota modifications using prebiotics, probiotics, postbiotics, feed additives, and vaccination. While some vaccines have proven to be effective in research settings, no commercial vaccines are currently available. Because no single strategy can effectively combat *Campylobacter*, integrating multiple approaches, such as improved biosecurity measures, immunization, and dietary modifications, may provide a solution for reducing *Campylobacter* loads in poultry. Embracing a “One Health” approach, gaining a deeper understanding of *Campylobacter* pathophysiology, advancing vaccine technology, and implementing holistic farm management practices will be essential for the sustainable control of Campylobacter and for reducing the risk of human campylobacteriosis.

## 1. Introduction

*Campylobacter* is one of the major causes of bacterial gastroenteritis in the United States [1,2]. Each year, an estimated 1.5 million people in the United States contract *Campylobacter* infections [3]. The primary source of these infections is raw or undercooked chicken meat containing high loads of *Campylobacter* originating from the chicken’s digestive tract [4,5,6]. The two major species responsible for human infections are *Campylobacter jejuni* and *Campylobacter coli* [7]. Apart from causing gastroenteritis, *C. jejuni* is linked to about one-third of Guillain-Barré Syndrome (GBS) cases in humans [8,9,10]. GBS is an immune-mediated peripheral nerve disease characterized by symmetrical ascending weakness that can progress to paralysis, accompanied by hyporeflexia and areflexia [11,12]. Thermophilic *Campylobacter* species, mainly *C. jejuni* and *C. coli,* are commonly found in wild birds and domestic poultry [13,14,15,16]. Some farms worldwide have reported *Campylobacter* prevalence rates as high as 100%, particularly among birds that have reached marketable age. Both *C. jejuni* and *C. coli* have adapted to the avian gastrointestinal tract (GIT). Despite widespread intestinal colonization (up to 10^9^ colony-forming units or CFU/g of cecal content), *Campylobacter* is often regarded as a commensal in birds, causing little to no overt illness [4,17,18,19]. However, recent studies have shown that *Campylobacter* spp. can lead to significant infections and elicit immune responses [20,21,22,23]. Following intestinal infection by *Campylobacter* in chickens, cytokine responses that drive humoral, adaptive, and Th17 responses have been observed [21,24,25]. Additionally, the newly emerged species, *Campylobacter hepaticus*, causes spotty liver disease (SLD) in layer hens, which is most prevalent during peak production stages [26,27,28].

Fluoroquinolones and macrolides were widely used in the past in animals for growth promotion and infection control purposes. They have also been prescribed as supportive treatments for human *Campylobacter* infections. However, this widespread use in food animals is believed to have significantly contributed to the development of antimicrobial resistance (AMR) against these antibiotics [29,30,31]. The emergence of AMR has restricted effective antibiotic treatment options for *Campylobacter* infections [31,32,33]. Consequently, growing concerns regarding AMR and food safety have led to bans on the use of medically important antimicrobials in food production systems for nontherapeutic purposes, driving an urgent search for alternative strategies that focus on *Campylobacter* control and prevention at the poultry farm level [33,34,35,36,37]. Achieving *Campylobacter* prevention in farm settings is quite challenging due to following reasons: (i) the ubiquitous nature of *Campylobacter*, (ii) multiple transmission routes, (iii) the low infection dose required for human illness, and (iv) the delayed detection of *Campylobacter* colonization or spread in birds [38,39,40,41]. Despite these challenges, quantitative microbial risk assessment studies have shown that a 1–2 log reduction in the level of *Campylobacter* in broiler chicken intestines can significantly impact relative risk reduction, achieving a decrease of 44–95% [42]. The incidence of campylobacteriosis through chicken meat can be reduced 30 times by introducing a 2 log reduction in the number of *Campylobacter* spp. in chicken carcasses [43]. Therefore, control of human *Campylobacter* infections is feasible through consistent application of safe practices from farm to fork.

*Campylobacter* control strategies can be broadly divided into two main categories: preharvest and postharvest strategies [44,45]. Preharvest strategies are measures and interventions to control *Campylobacter* at the farm level. These strategies mainly focus on reducing *Campylobacter* colonization and preventing its introduction and spread in the environment [35,46,47]. Preharvest strategies can be further divided into three categories: (i) reduction in environmental exposure through biosecurity measures; (ii) reduction of *Campylobacter* colonization in bird intestines by improving host resistance via competitive exclusion, vaccination, and host genetic selection; and (iii) use of alternatives to antibiotics to mitigate *Campylobacter* colonization in birds [48]. Postharvest interventions include carcass decontamination, antimicrobial treatment for poultry processing, cold chain management, and consumer education [49,50,51,52,53,54,55]. However, most of these interventions are ineffective when used alone and when shown to be effective, the products are not commercially available. 

While vaccines have shown promising results in the prevention of various poultry diseases, and many studies have tested numerous vaccine candidates, no commercial vaccines are currently available to prevent or reduce *Campylobacter* colonization in chickens. A multifaceted approach that combines two or three strategies is essential for preventing and controlling *Campylobacter* colonization in poultry. This comprehensive review explores the current state of preharvest approaches to mitigate *Campylobacter* colonization in poultry, with a special emphasis on vaccination strategies against *Campylobacter* spp.

## 2. *Campylobacter* in Broilers—Biology and Public Health Impact

*Campylobacter* spp. are Gram-negative, motile, slender, comma-shaped or spiral-shaped, non-spore-forming bacteria. They grow strictly under anaerobic-to-microaerophilic conditions and are nutritionally fastidious. Their length ranges from 0.5 to 5 µm, and the width ranges from 0.2 to 0.9 µm [7,56]. There are more than 57 *Campylobacter* spp. under the genus *Campylobacter* (https://lpsn.dsmz.de/genus/Campylobacter accessed on 8 August 2025). They colonize the intestines of many warm-blooded hosts, including humans; however, avian species are more favorable as commensal colonizers [57]. In humans, *Campylobacter* causes gastroenteritis, which can sometimes lead to complications such as GBS, irritable bowel syndrome (IBS), and reactive arthritis [56]. In the United States, *Campylobacter* is one of the major causes of gastroenteritis with approximately 1.3 million cases leading to economic costs ranging from USD 1.3 to USD 6.8 billion [58]. Generally, self-limited diarrheal illness lasts for about 5–7 days, but elderly people with immuno-compromised status are at a high risk for mortality, morbidity, and prolonged illness [7].

*C. jejuni* and *C. coli* are the major *Campylobacter* species associated with human illness. Humans acquire infections through fecal–oral transmission from infected animals and food products [59,60]. Avian species, especially chickens, account for an estimated 50–70% of reported *Campylobacter* infections in humans [61]. When chickens carry *Campylobacter* in their intestines, their meat may become contaminated during slaughter and processing [62]. As few as 500–800 CFU of *C. jejuni* are sufficient to cause infection, implying that bacteria do not need to multiply to cause disease [63,64]. 

*Campylobacter* can colonize the mucus of the small intestine and ceca of chickens, sometimes at very low densities such as 40 CFU [65]. Once colonization occurs, bacteria rapidly reach high numbers in the cecal contents [66,67,68]. Chickens are coprophagic, meaning that they consume feces, which allows the rapid spread of *Campylobacter* rapidly throughout the flock. Once *Campylobacter* colonization is detected in a flock, most birds in the flock typically become colonized within days [69,70,71,72]. There is a direct correlation between *Campylobacter* prevalence in chickens and the likelihood of human *Campylobacter* infections. Therefore, reducing the prevalence of *Campylobacter* in chicken flocks has the potential to significantly decrease human infections [73]. This approach has been quite successful in countries such as Denmark and Iceland due to coordinated national action, targeted biosecurity interventions (e.g., fly screens), and systematic surveillance programs that track progress using rigorous indicators [74,75].

## 3. Overview of Preharvest Control Strategies

**Figure 1:**
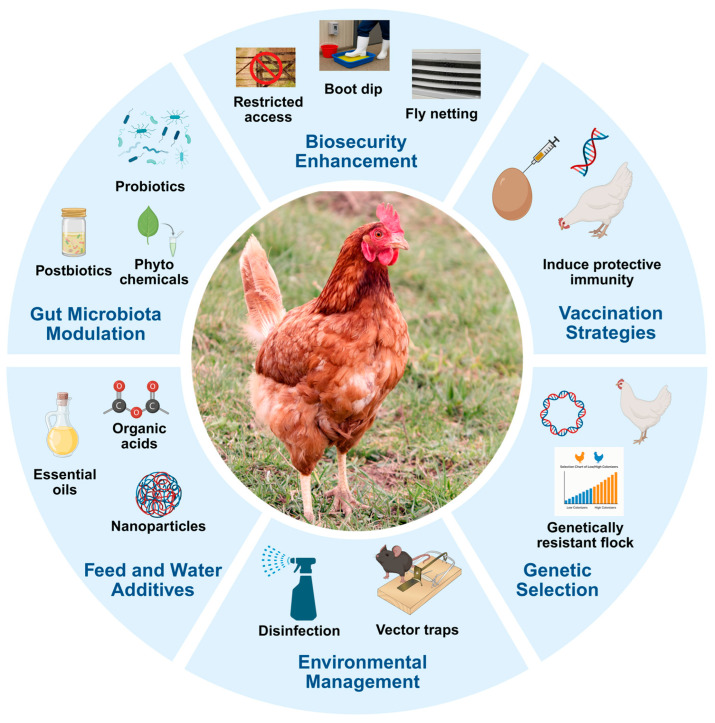
Preharvest intervention strategies to control *Campylobacter* in poultry (created in BioRender).

Various non-antibiotic interventions have been tested to reduce the *Campylobacter* colonization in poultry during the preharvest phase (Figure 1). These include biosecurity measures, prebiotics, probiotics, postbiotics, feed additives, bacteriophage therapy, vaccination, and genetic selection for resistant chicken strains.

### 3.1. Biosecurity Measures

**Figure 2:**
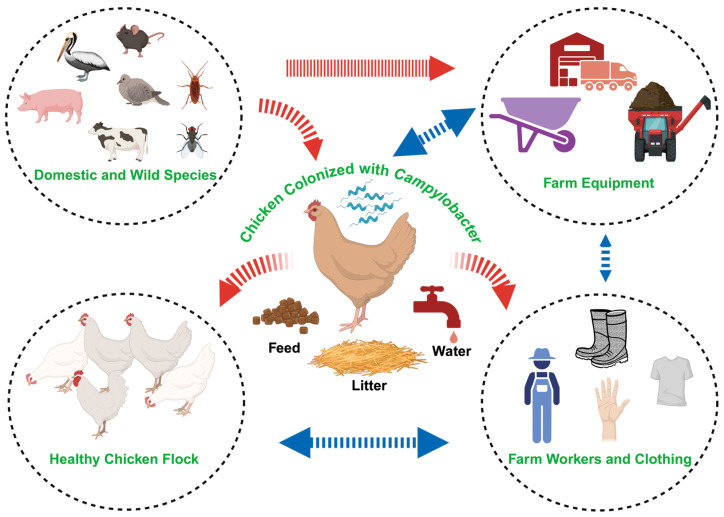
On farm transmission cycle of *Campylobacter* in poultry production (created in BioRender).

Biosecurity is crucial for keeping *Campylobacter* out of animal flocks, as it acts as the primary defense against this pathogen [47,76]. In poultry, the transmission route of *Campylobacter* is horizontal (Figure 2). There are no known reports on the vertical transmission of *Campylobacter* spp. Potential sources of *Campylobacter* into farm include domestic and wild animals, farm equipment, and contaminated litter, feed, and water, as well as potential transmission from infected birds [77,78,79,80,81]. The poultry house interior environment showed a lower prevalence of *Campylobacter* in air/ventilation samples (6%), pests (5%), litter (3%), water samples (2%), and feed (rarely), in descending order of *Campylobacter* prevalence rates. The external environment of the poultry house showed 14% prevalence, with 67% and 14% prevalence in domestic animals and their excreta, respectively. The transport equipment used for live haul, including trucks (44%) and crates (22%), showed different prevalence rates of *Campylobacter* [78]. Although implementing strict biosecurity measures can be challenging, they are fundamental in preventing initial colonization. Many interventions primarily focus on reducing *Campylobacter* levels after they are already present, but biosecurity protocols help prevent them from entering the farm in the first place. The effectiveness of biosecurity is greatly enhanced when combined with other successful strategies [82,83].

#### 3.1.1. Managing Human Entry and Hygiene to Prevent Contamination

*Campylobacter* bacteria are frequently found in agricultural workers, farm managers, and truck drivers. To reduce the number of *Campylobacter*-positive flocks, it is recommended to limit human traffic by restricting unnecessary movements of people and minimizing visitors to farms and animal housing. The following practices can help reduce the entry of *Campylobacter* through humans: (i) Enforce the use of personal protective equipment (PPE): PPE should be mandatory for anyone making essential visits to the farm. (ii) Maintain dedicated hygiene measures: Regularly cleaned and disinfected footwear and clothing, specifically, should be designated for each poultry house. This practice helps create a stronger hygiene barrier. (iii) Promote hand hygiene: Handwashing stations should be accessible at all entry points to poultry houses. Everyone must be instructed to thoroughly sanitize their hands for 15–20 s both before entering and after leaving the animal housing. (iv) Avoid high-risk activities: To significantly reduce contamination risks, it is important to avoid unnecessary movements of people, particularly during high-risk activities such as thinning [47,84]. Despite having clear guidelines, biosecurity protocols are often not followed meticulously. To achieve a greater impact, comprehensive training, education, and consistent monitoring are essential to ensure adherence to best practices [83,85].

#### 3.1.2. Equipment and Vehicle Sanitation

The movement of vehicles and equipment between houses or farms poses a significant risk of *Campylobacter* transmission. It is not advisable to transfer the equipment unless it is properly cleaned. *Campylobacter* can survive longer periods on equipment surfaces, by entering a viable but non-culturable state (VBNC), which makes it more difficult to eliminate from the environment and enables them to survive under a variety of stress conditions [86,87]. Residual organic matter can further protect *Campylobacter* from standard, protecting the standard washing procedures, allowing them to persist in the environment and act as a continuous source of contamination [38]. Therefore, it is necessary to employ effective sanitation and disinfection methods to prevent the spread of *Campylobacter*. This process involves more than just washing; it requires a multistep approach that includes dry cleaning, wet cleaning, disinfection, and drying [82].

#### 3.1.3. Pest and Wildlife Control

Animals, including cattle and poultry, are known reservoirs of *Campylobacter*, which has been isolated from the intestinal tracts of various animals and birds [88,89,90,91]. Wildlife serves as an amplifying host, exhibiting a high pathogen shedding capacity and playing an important role in transmission [77]. Wild birds are particularly important because they can spread *Campylobacter* across different geographical areas because of their ability to fly over large distances [92,93]. In addition to domestic and wild animals, birds, rodents, and insects have been shown to transmit *Campylobacter* [94,95,96,97,98]. To control its spread, robust vector control programs should be implemented targeting wild animals, rodents, and insects. Comprehensive integrated pest management programs can help eliminate pest attractants and breeding sites from the surrounding environment. Effective strategies include rodent-proofing measures, targeted larvicides for improved litter management to exclude and control flies, and bird-proof sealing to deter wild birds [44,47].

### 3.2. Probiotics, Prebiotics, and Postbiotics

In the post-antibiotic era, there is a growing interest in probiotics, prebiotics, and postbiotics as effective dietary interventions [99]. Probiotics are non-pathogenic live organisms that confer health benefits to the host when consumed in adequate amounts [100]. Common probiotic microorganisms belong to the genera *Lactobacillus*, *Bifidobacterium*, *Saccharomyces*, *Bacillus*, *Streptococcus*, and *Enterococcus* [101,102,103]. They positively influence the host through various mechanisms, such as improved intestinal barrier function, immunomodulation, and production of neurotransmitters [104]. Probiotic supplementation in chicken diets helps maintain intestinal homeostasis, eliminates pathogenic bacteria through competitive exclusion, and stimulates the secretion of important digestive enzymes, such as phytases, amylases, and proteases, thereby improving feed utilization efficiency [105,106,107,108,109,110,111]. Chickens are monogastric animals, with a single-chamber stomach divided into two distinct regions: the proventriculus (glandular stomach) and the gizzard (muscular stomach), followed by the small and large intestines [112]. The entire GIT functions in close symbiosis with the resident microbiota to aid in digestion and nutrient absorption, while also playing a crucial role in maintaining health and optimizing production by regulating physiological processes [113,114,115]. The chicken gut microbiota is highly complex and is dominated by bacteria, with over 600 different bacterial species identified [116]. While bacterial diversity varies throughout the GIT, the cecum is the most densely colonized region. The cecum plays a key role in pathogen colonization [117,118]. Under uncertain conditions, an imbalance in normal gut microbiota can promote the growth of opportunistic and pathogenic bacteria, thereby disrupting gut health. Probiotics can help in this situation by restoring the beneficial gut microflora and preserving gut integrity [117,119,120,121].

Prebiotics are non-digestible food components, generally metabolized by specific bacteria, and provide beneficial effects on the host [118,122]. They help increase the abundance of beneficial microorganisms, such as bifidobacteria and lactobacilli, and improve gut metabolic activity, resulting in the production of a series of metabolites that favor the maintenance of gut health [118,119]. Prebiotics consist of monomers derived from common sugars, including glucose, galactose, fructose, and xylose. Widely studied examples are insulin, fructooligosaccharides (FOS), isomalto-oligosaccharides (IMO), and galactooligosaccharides (GOS). Postbiotics are functional bioactive molecules produced during the metabolic processes of probiotics, which confer health benefits to the host [119,123]. Unlike live probiotics, postbiotics offer a safer and more stable alternative by mitigating the key limitations that impede the broader application of probiotics in commercial settings, such as the risk of antimicrobial resistance, poor thermal stability, and potential for expressing virulence factors [124]. According to the International Scientific Association for Probiotics and Prebiotics (ISAPP) [125], postbiotics are composed of inactivated microbial cells, bacteriocins, cell-free supernatants, exopolysaccharides, and short-chain fatty acids [126]. A growing body of in vitro and in vivo evidence indicates that postbiotics enhance gastrointestinal health by promoting beneficial bacterial populations, modulating host immune responses, and supporting intestinal barrier integrity [127,128,129,130].

### 3.3. Bacteriophage Application in Campylobacter Control

The application of bacteriophages as a biocontrol strategy has been investigated for controlling food-borne pathogens (e.g., *Listeria*, *Salmonella,* and *E. coli* O157:H7) [131]. Bacteriophages are viruses that infect bacterial cells and have demonstrated potential as therapeutic agents against bacteria. Bacteriophages used in these treatments are specific to bacteria. For instance, certain *Salmonella* bacteriophages (ST27, ST29, and ST35) are specific to the TolC receptors of *Salmonella* serovars. The binding specificity of bacteriophages to bacteria determines their host range. Upon entering a bacterial cell, bacteriophages generally undergo either a lysogenic or lytic cycle. Bacteriophages utilize the host machinery to produce their progeny. Because of the low risk of phage transduction and rapid lysis activity, lytic phages are preferred as therapeutic targets over lysogenic phages. It is estimated that a 2 log CFU reduction in *Campylobacter* levels in poultry intestines is sufficient to reduce the occurrence of human campylobacteriosis associated with poultry by 30-fold [132]. Chinivasagam et al. used a cocktail of bacteriophages to control *Campylobacter* in a commercial broiler setting. One of the farms involved in the trial achieved a 1–3 log_10_ CFU/g significant reduction in *Campylobacter* loads in the ceca of 47-day-old broiler chickens compared with the control group. Another study showed a non-significant 1.7 log_10_ CFU/g reduction in *Campylobacter* [133]. In a recent study, treatment of *Campylobacter*-colonized broiler chickens with a cocktail of two virulent *Campylobacter* bacteriophages, CP20 and CP30A, resulted in a significant 2.4 log_10_ CFU g^−1^ reduction in *Campylobacter* levels for two days post-treatment in infected chickens compared to mock-treated controls [134].

### 3.4. Feed Additives

In poultry production, organic acids such as acidifiers (e.g., formic, butyric), essential oils (EOs) (e.g., thymol, carvacrol), and diverse plant extracts (phytogenic) are increasingly utilized as alternatives to antibiotic growth promoters. These substances play an important role in enhancing intestinal health, primarily by modulating the gut microbiota [135,136]. Organic acids are naturally produced during the metabolism of various animal feed components. They help lower intestinal pH, thereby inhibiting the proliferation of pH-sensitive enteric pathogens such as *Salmonella* and *E. coli*. This acidic environment allows the undissociated form of these acids to pass across bacterial cell membranes, leading to intracellular acidification, disruption of metabolic processes, and eventual bacterial lysis, while simultaneously fostering the growth of beneficial acid-tolerant bacteria, such as *Lactobacillus* and *Bifidobacterium* [137,138,139]. Organic acids also aid in the absorption of vital micro- and macro-minerals, such as calcium, magnesium, and zinc [137]. EOs are strong antioxidants and antibacterial agents [140]. These substances are rich in lipophilic phenolic compounds capable of disrupting bacterial cell membrane integrity, increasing permeability, and causing leakage of cytoplasmic contents, thereby contributing to their broad-spectrum antimicrobial effects against pathogens such as *Clostridium perfringens* and *E. coli* [141]. EOs can also neutralize free radicals and exhibit potential antioxidant properties [142,143]. Plant extracts are generally regarded as safe, and many can be consumed as food [144,145]. These extracts contain a complex array of bioactive compounds, including flavonoids, tannins, and alkaloids. They exhibit multifaceted mechanisms, such as direct antimicrobial, anti-inflammatory, and immunomodulatory properties that strengthen the gut barrier and can stimulate digestive secretions, collectively shifting microbial communities towards a healthier and more diverse microbial profile that favors commensal bacteria and optimizes nutrient utilization [146,147,148,149]. For example, herbal compounds like tryptanthrin have been shown to significantly reduce *Campylobacter* colonization in vitro and in vivo [150].

### 3.5. Vaccination—A Targeted Approach

Vaccination is a proven strategy for the prevention and control of bacterial and viral infections. Compared to other management strategies, it offers distinct advantages with respect to public health impact and long-term sustainability [151,152]. Currently, no commercial vaccine is available to protect chickens from colonization [151,153,154]. Although vaccines are not 100% successful in preventing *Campylobacter* colonization in hens, they have been shown to be more effective than previously reported methods. Better protection could potentially be obtained by combining immunization with additional preharvest strategies [152,155,156].

**Figure 3:**
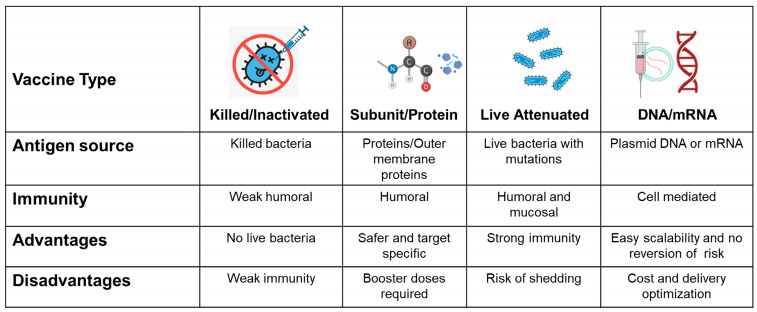
Major types of vaccine strategies tested to control bacterial infections in poultry (created in BioRender).

Various vaccines against *Campylobacter* in poultry have been tested, relying on different antigen sources and immune mechanisms, each with unique advantages and limitations. Killed or inactivated vaccines, prepared from whole bacteria, are considered to be safe and eliminate the risks associated with live organisms; however, they typically induce only weak humoral immune responses [156]. Subunit or protein vaccines, based on outer-membrane proteins or antigens purified using recombinant DNA technologies, are safer and more target-specific, although they often require booster doses to sustain effective immunity [157,158]. Live-attenuated vaccines, generated from genetically modified bacteria with reduced virulence, stimulate both humoral and mucosal immunity and can provide strong protection, but they carry the potential risk of shedding the vaccine strain [159,160]. Finally, DNA or mRNA vaccines, which deliver genetic material encoding specific antigens, primarily induce cell-mediated immunity and offer advantages, such as scalability and no risk of reversion; however, their cost and delivery challenges remain major limitations [161,162]. Figure 3 illustrates the different vaccine strategies that have been evaluated experimentally or implemented in practice for the prevention and control of bacterial infections in poultry. These include killed/inactivated vaccines, subunit vaccines, live attenuated vaccines, DNA vaccines, and mRNA vaccines, each with distinct advantages and disadvantages.

#### 3.5.1. Types of Poultry *Campylobacter* Vaccines

##### Subunit Vaccines

Subunit vaccines use bacterial components instead of whole bacteria to trigger an immune response. They generally offer advantages over attenuated and killed vaccines in terms of lower risk of reverting to virulence, enhanced safety, targeted immunity, and better compatibility with adjuvants. Despite these advantages, the development of effective subunit vaccines remains a challenge. One major difficulty is identifying suitable antigens capable of protecting different *Campylobacter* species or even serotypes and strains within the same species. In addition, providing robust immunity to protect broiler chickens with a shorter lifespan requires an optimized delivery method. To date, several antigens tested as subunit vaccines have shown modest-to-significant results [157,159,163].

##### Live-Attenuated Vaccines

Live attenuated vaccines are live bacteria that result from reduced virulence/pathogenicity, but are capable of generating adequate long-lasting immunogenicity while activating both adaptive and innate immune responses [151,164]. Live attenuated vaccines tested against *Campylobacter* include heterologous bacterial vectors that transport *Campylobacter* antigens and strains with mutated oxidative stress defense antigens [151,155]. Another approach to live attenuated vaccines is to use *E. coli* to deliver glycoconjugated antigens, thus improving the vaccine performance [165]. These vaccines offer more advantages than killed and subunit vaccines by providing long-lasting immune responses, including mucosal immunity. Despite these advantages, the risk of reverting to virulent forms and interference with material antibodies in young chickens are major concerns regarding subunit vaccines [166]. Environmental contamination through the shedding of vaccine strains is an additional concern, making it crucial to select a strain that guarantees both safety and immunogenicity without posing any environmental biohazard risks [166,167].

##### Inactivated/Killed Vaccines

The concept behind inactivated or killed vaccines is that, after undergoing physical or chemical treatments, bacteria retain their immunogenic antigens, which can still elicit an immune response [168]. However, studies evaluating inactivated or killed vaccines have shown limited success [169,170,171]. A major challenge with killed poultry vaccines is identifying an effective adjuvant to boost the immune response [172]. Additionally, inactivated/killed vaccines do not generate the mucosal immune response essential for reducing *Campylobacter* colonization. These vaccines must be administered via a parenteral route, prohibiting mass administration and making them economically not feasible [166,173,174].

##### DNA and mRNA Vaccines

Genetic vaccines represent a significant advancement in the field of vaccinology [175,176,177]. These vaccines do not require a live vector for delivery; they use host–cell mechanisms to produce antigens. Genetic vaccines primarily consist of DNA or mRNA, which are taken up by cells and translated into proteins [162]. Various DNA vaccines based on flagellin, outer-membrane protein, and prime-boost vaccines have been investigated for *Campylobacter* control [178,179,180]. DNA and mRNA vaccines are generally safer to administer because they do not involve the risks associated with live pathogens [177,181]. They are capable of eliciting both humoral and cellular immune responses, even in the presence of maternal antibodies [182,183]. Although genetic vaccines demonstrated a high rate of success, optimizing delivery and ensuring efficient cellular uptake are critical to their overall effectiveness. In particular, mRNA vaccines delivered via lipid nanoparticles require further refinement in both formulation and storage, as current methods are not cost-effective for mass immunization [184,185].

#### 3.5.2. Challenges in *Campylobacter* Vaccine Development

##### *Campylobacter* Properties

Pan-genome analyses of *Campylobacter* have revealed extensive genomic variability, highlighting its highly diverse nature at the genome level [186,187,188,189]. This significant genetic diversity indicates that a vaccine targeting only one or a few strains may not be effective against many circulating *Campylobacter* strains in the field. Another challenge is the phase variation phenomenon, which allows bacteria to swiftly adapt to their new surroundings and effectively colonize and survive during the host immune response [190,191,192]. Through phase variation, bacteria can generate new subpopulations with distinct phenotypes without undergoing overall changes in their genetic content [192,193,194]. In *Campylobacter*, more than 30 genes, including those encoding key cell surface components, such as lipooligosaccharides, capsular polysaccharides, and flagellin, are differentially regulated in response to external environmental factors. This phase variation leads to the expression of different versions of surface antigens, which can make vaccines ineffective since the immune response produced by the vaccine may no longer recognize the altered antigens. Consequently, polymorphisms arising from phase variation present a challenge for developing a single vaccine effective against all relevant bacterial forms. Even the vaccines that initially provide protective immunity may eventually lose their effectiveness as the bacterial population dynamically changes its antigen profile [195,196].

##### Host Factors Influencing Vaccinal Immunity

One of the major hurdles in *Campylobacter* vaccine development is the poor understanding of *Campylobacter* infection immunobiology [152,197,198]. Typically, newly hatched chicks are *Campylobacter*-free, and maternal antibodies provide initial protection by delaying the start of colonization [199,200,201,202]. Vaccination of breeder hens with bacterin and subunit vaccines resulted in chicks possessing anti-*Campylobacter* antibodies in their blood and mucus, offering some protection, although this protection waned after approximately two weeks [203,204]. Notably, *Campylobacter* colonization usually begins at approximately three weeks of age, a timeframe that coincides with a decrease in maternal antibody levels [204,205,206]. In addition to this complexity, the mucosal immune system of chicks does not fully mature until around seven weeks, which is after the typical six-week market age for broilers [24,25,207]. This delayed immune maturation is further supported by studies on antibody-associated clearance in bursectomized birds, which indicate that adaptive immune responses develop after approximately six weeks, suggesting that achieving effective immune-based protection is more feasible in adult birds [160,161,199,208,209].

The mucous layers of the lower digestive tract are colonized by *Campylobacter* without provoking any notable immune response from the host [210]. In contrast, effective vaccines must stimulate a strong intestinal mucosal immunity to prevent *Campylobacter* colonization and infection [207]. Most injectable vaccines do not produce adequate immunity because *Campylobacter* remains confined to the intestinal lumen and does not cause significant tissue invasion or systemic infection capable of triggering robust mucosal immune responses. In addition, the anatomical features of the chicken immune system also present several obstacles to effective vaccination. The Bursa of Fabricius is a specialized lymphoid organ essential for the development of B cells and antibody production; however, it undergoes regression with age, reducing its immunological capacity over time [211]. Unlike mammals, chickens lack encapsulated lymph nodes, which are the key sites of antigen presentation and the initiation of adaptive immune responses. Consequently,, the diffuse and aggregated secondary lymphoid tissues within the gut-associated lymphoid tissues (GALT) play a central role in vaccine-induced immunity [212,213]. In particular, the cecal tonsils and Peyer’s patches of the GALT, serve as major inductive sites for mucosal immunity in poultry [214,215]. Microfold (M) cells overlying these lymphoid follicles facilitate antigen uptake and transfer to underlying immune cells [216,217]. Therefore, effective vaccines that target GALT and stimulate mucosal immunity are required for effective *Campylobacter* control [218,219].

##### Administration and Management of Vaccines

Although small-scale laboratory experiments have shown success, *Campylobacter* vaccines do not yield the same effectiveness in field conditions. The diverse nature of poultry rearing systems, spanning from small-scale backyard operations to large-scale commercial enterprises, presents a significant challenge for the implementation of a standardized and universally effective vaccination protocol [67]. In controlled laboratory settings, each bird receives a precisely measured vaccine dose, which is impractical in field settings. To enable practical and cost-effective scaling up for larger flocks, mass vaccine administration techniques such as in ovo, water, or spray application systems are employed. While in ovo vaccination has been highly successful for certain diseases such as Marek’s disease, it cannot be considered universally applicable due to both host-related factors (e.g., the immature immune system and potential impacts on embryonic safety) and antigen-related factors (e.g., protein stability) [220,221,222]. These techniques often result in irregular immune responses and varying rates of vaccine uptake [223].

#### 3.5.3. Positive Outcomes and Promising *Campylobacter* Vaccine Candidates

Although the primary focus of this review is on vaccine studies reporting substantial and statistically significant reductions in *Campylobacter* colonization, Table 1 provides an overview of all poultry *Campylobacter* vaccine studies conducted to date. While no commercial *Campylobacter* vaccine for poultry is currently available, several approaches have shown significant reductions in *Campylobacter* colonization in chicken intestines. Live vector vaccines, recombinant proteins, DNA-based constructs, and conjugate vaccines stand out as approaches demonstrating promising efficacy, albeit to varying degrees. These findings highlight the potential for optimizing and developing scalable vaccination strategies in the future. Among live vector vaccines, oral administration of live attenuated *Salmonella* Typhimurium strain expressing *C. jejuni* CjaA (*C. jejuni* antigen A) at day 1 and day 14 of age or *S*. Typhimurium expressing Dps (DNA-binding protein from starved cells) at day 3, day 10, and day 16 reduced *Campylobacter* colonization by 1.4 log_10_ and 2.92 log_10_, respectively, following a challenge with *C. jejuni* [160,224]. Similarly, a live attenuated *S*. Typhimurium expressing linear peptides of *C. jejuni* antigens Cj0113 produced even more striking results, yielding a 4–4.8 log_10_ reduction in *Campylobacter* loads in the ileum and, in some cases, driving bacteria to undetectable levels [161]. When chickens were vaccinated with *Lactococcus lactis* expressing CjaA, the vaccinated chickens showed a 2.05–2.35 log_10_ reduction in *Campylobacter* 5 days post-challenge [163]. However, when CjaA was expressed in *Eimeria tenella* and administered orally to White Leghorn chickens as a transfected parasite vaccine, it only resulted in a one-order reduction in *Campylobacter* colonization [225].

Autogenous vaccines, based on whole-cell preparations tailored to specific farms, have achieved nearly 50% reduction in *Campylobacter* colonization and have also been associated with decreased bacterial survival meat surfaces [226]. In addition to these, subunit and recombinant protein vaccines constitute a major category of poultry *Campylobacter* vaccines. While various *Campylobacter* proteins have been explored as vaccine candidates, only a limited number have yielded promising results. For example, vaccination of White Leghorns at day 1 and day 14 with a recombinant FliD (flagella capping protein) administered subcutaneously led to 2 log_10_ reduction in *Campylobacter* counts following *C. jejuni* challenge [158]. Similarly, proteins such as CjaA and Dps have demonstrated varying levels of efficacy when delivered via live bacterial vectors or *E. tenella,* however, their purified forms alone failed to reduce *Campylobacter* loads in chicken intestines [160,163,224,225]. In another study, chickens vaccinated intramuscularly at day 6 and day 16 with CadF (adhesin), FlpA (adhesin), and FlaA (flagellin) peptides showed reduced cecal *C. jejuni* loads a reduction in the number of *C. jejuni* loads in the ceca compared to unvaccinated challenged controls [157]. Some *Campylobacter* proteins, when conjugated to carrier proteins or designed as hybrid proteins, have yielded particularly encouraging results. For instance, immunization of White Leghorn chickens with keyhole limpet hemocyanin (KLH) conjugated with enterobactin via the intramuscular route resulted in a 3–4 log_10_ reduction in *C. jejuni* in the ceca of vaccinated chickens compared to unvaccinated controls [227]. Likewise, a hybrid protein combining *C. jejuni* flagellin (FlaA) with the B-subunit of the labile toxin (LT-B) from *Escherichia coli*, significantly reduced the number of *Campylobacter* colonized chickens two-weeks post-challenge [228]. In another study, a fusion protein of CadF-FlaA-FlpA, termed the “Trifecta vaccine”, was shown to decrease intestinal bacterial loads in vaccinated chickens in a challenge study [157].

Several studies have also explored the use of non-live carriers—such as liposomes, nanoparticles, and Gram-positive Enhancer Matrix (GEM) particles of *Lactococcus salivarius*—to deliver *Campylobacter* antigens and prevent *Campylobacter* colonization in chickens. For example, in ovo delivery of the hybrid protein rCjaAD (CjaA presenting CjaD epitopes on its surface) via GEM particles or liposomes at embryonic day 18 protected chickens from *C. jejuni* challenge [229]. With the advent of nanoparticle-based vaccines, multiple approaches have been investigated to develop an efficacious *Campylobacter* vaccine for chickens. In one study, intranasal administration of chitosan-DNA nanoparticles carrying *flaA* reduced *Campylobacter* loads in both the large intestine and cecum [230]. Similarly, four antigens of *C. jejuni* identified through reverse vaccinology (YP_001000437.1, YP_001000562.1, YP_999817.1, and YP_999838.1) significantly reduced cecal colonization of *Campylobacter* when administered intramuscularly as DNA vaccines combined with unmethylated CpG oligodeoxynucleotide (ODN) followed by intramuscular administration of those antigens as recombinant proteins a week later [231]. Another study demonstrated that oral administration of poly (D, L-lactic-co-glycolic acid) (PLGA) nanoparticle-encapsulated, CpG ODN, along with *C. jejuni* lysate, markedly reduced intestinal colonization of *C. jejuni* by enhancing the proliferation of specific microbial groups [232]. Likewise, oral gavage of recombinant hemolysin co-regulated protein (Hcp—a key component of the Type VI secretion system (T6SS)—entrapped in chitosan–sodium tripolyphosphate nanoparticles (CS-TPP NPs) achieved superior clearance of *Campylobacter* compared to subcutaneous delivery of the protein emulsified with incomplete Freund’s adjuvant [233].

In addition to protein antigens, *C. jejuni* capsular polysaccharide antigens (CPS) have also been evaluated for their efficacy as vaccines in chickens. In one study, the CPS-diphtheria toxoid conjugate vaccine (CPSconj), administered subcutaneously with either CpG or a squalene-based adjuvant, effectively reduced cecal colonization of *Campylobacter* in broiler chickens following a challenge with *C. jejuni* [234]. Other vaccines have been developed using protein–glycan coupling technology (PGCT), which employs *E. coli* harboring the *C. jejuni pgl* locus to glycosylate antigens. Notably, two intramuscular doses of a glycosylated *Pseudomonas aeruginosa* exotoxin A (G-ExoA) demonstrated superior efficacy in reducing *Campylobacter* colonization compared to the unglycosylated ExoA counterpart [235].

##### Autogenous Vaccines

A whole-cell autogenous vaccine targeting *Campylobacter* genes essential for extraintestinal survival was developed using a genomic tailoring approach. The progeny of broiler breeders that received the vaccine showed a nearly 50% decrease in *Campylobacter* isolates that colonized and carried extraintestinal survival genes, as well as a notable decrease in survival on meat surfaces. A logistic regression model estimated that the vaccine could successfully target 65% of the population of clinically relevant *Campylobacter* strains. This vaccine strategy is an effective approach to combating bacterial infections by specifically targeting bacterial lineages linked to infection and transmission risks within a larger commensal population [226].

##### Subunit Vaccines

Subcutaneous administration of 125 µg of the outer membrane (OMP) fraction of *C. jejuni* resulted in significantly lower *Campylobacter* levels in the cecal contents than the oral route of administration. When these outer membrane components were delivered subcutaneously via nanoparticles, *Campylobacter* was undetectable in the intestines. In contrast, 13% of chickens showed detectable intestinal *Campylobacter* levels following subcutaneous administration of non-encapsulated outer-membrane components. The serum IgA and IgY (IgG) responses appeared earlier and were higher in the groups that received the vaccine subcutaneously, with the nanoparticle-encapsulated OMP vaccine showing higher IgY and IgA titers in cloacal feces than the other OMP vaccine types. These findings indicate that subcutaneous delivery of OMPs, with or without nanoparticle encapsulation, effectively stimulates antibody production and significantly reduces *Campylobacter* colonization in the intestine [236]. Similarly, vaccination with chitosan/pCAGGS-*flaA* nanoparticles intranasally reduced the bacterial colonization by 2–3 log_10_ [230]. Furthermore, vaccination with recombinant peptides derived from CadF, FlaA, and a combined CadF-FlaA-FlpA protein of *C. jejuni* significantly lowered *Campylobacter* loads in the ceca, with median log_10_ reductions of 3.35 for CadF, 3.11 for FlaA, and 3.16 for the fusion protein [157].

##### Live Attenuated Vaccines

Vaccinating chickens with a modified *Salmonella* strain expressing the *cjaA* gene from *C. jejuni* stimulated the production of IgY and IgA antibodies against the outer surfaces of both *Salmonella* and *Campylobacter*. In contrast to the control group, in which all chickens were heavily colonized, only 15% of the vaccinated chickens had high levels of *Campylobacter* (above 10^3^ CFU/g) in their ceca [159]. Similarly, a *Salmonella* strain carrying the *dps* gene of *C. jejuni* demonstrated a 2.5 log reduction in *Campylobacter* levels following experimental infection [224]. Oral delivery of an *E. coli* strain that produces *C. jejuni* N-glycan resulted in 65% protection against *Campylobacter* colonization, whereas all unvaccinated chickens became colonized. Combining the N-glycan vaccine with probiotics, such as *A. mobilis* or *L. reuteri,* enhanced weight gain, IgY antibody production, and overall vaccine efficacy [165].

##### DNA Vaccine

Four novel vaccine candidates discovered using reverse vaccination technology demonstrated a significant decrease in the cecal burden of *Campylobacter* in Ross broiler chickens when administered as DNA vaccines. These candidates achieved a 4.2 log_10_ CFU/g decrease in *Campylobacter* load, which could potentially translate into a 76–100% reduction in the risk of human campylobacteriosis. by However, these promising results have proven difficult to reproduce consistently, necessitating further investigation to develop a reliable and broadly effective vaccine [43,231,237,238].

**Table 1:** Summary of the vaccine approaches investigated for poultry *Campylobacter*.

Vaccine	Chicken Breed (Chicken Type)	Age at Vaccination	Vaccination Regimen	Challenge		Reduction in Levels (Mean log_10_ CFU/Gram) of *Campylobacter*	Reference
				Age	Strain (Dose)		
Live attenuated *Salmonella* vaccine expressing CfrA or CmeC proteins	Cornish × Rock (broiler)	Day 7	Oral administration of 200 μL of *Salmonella* (1 × 10^9^ CFU/mL) expressing CfrA or CmeC	Day 28	*C. jejuni* NCTC 11,168 (2 × 10^3^ CFU/bird)	No significant reduction	[155]
Nanoparticle-encapsulated OMPs of *C. jejuni* 81–176	Not specified	Day 7 and Day 21	Oral administration of 25 or 125 µg of nanoparticle-encapsulated OMPs or OMPs alone	Day 35	*C. jejuni* 81–176 (2 × 10^7^ CFU/bird)	No significant reduction	[236]
			Subcutaneous administration of 25 or 125 µg of nanoparticle-encapsulated OMPs or OMPs alone				
Live *Salmonella* Typhimurium Δ*aroA* strain expressing CjaA of *C. jejuni*	Light Sussex (broiler)	Day 1 and Day 14	Oral gavage of 0.3 mL of stationary phase culture (1 × 10^8^ CFU/mL)	Day 28	*C. jejuni* M1 (1 × 10^7^ CFU/bird)	Significant 1.4 log_10_ CFU/g reduction	[160]
Purified recombinant CjaA	Light Sussex chickens (broiler)	Day 1 and Day 15, or Day 15 and Day 29	Subcutaneous administration of 14 μg of rCjaA with TiterMax adjuvant	Day 29/Day 44		No significant reduction	
Autogenous poultry vaccine	Ross (broiler)	14 and 18 weeks of age	Intramuscular administration of 0.5 mL of oil-based autogenous vaccine	Not a challenge study	Measured natural colonization	No significant reduction	[226]
FliD and FspA	White Leghorn (layer)	Day 1 and Day 14	Subcutaneous administration of 4.3 × 10^10^ moles of each recombinant protein, FliD and FspA, with TiterMax Gold adjuvant	Day 28	*C. jejuni* M1 (1 × 10^7^ CFU/bird)	2 log_10_ CFU/g in reduction with FliD (statistically significant)	[158]
*Eimeria tenella*-expressing CjaA	White Leghorn (layer)	Group 1: Day 1 Group 2: 1/3/7/20	Oral administration of 100, 500, 3000, and 5000 fourth-generation *CjaA*-transfected parasites	Day 28	*C. jejuni* 02M6380 (1 × 10^5^ CFU/bird)	One-order reduction (statistically significant)	[225]
FlpA with ten N-heptasaccharide glycan moieties	White Leghorn (layer)	Day 0 and Day 14	Subcutaneous administration of 100 μg of FlpA with TiterMax Gold or the molar equivalent of FlpA-10 × GT in 100 µL	Day 28	*C. jejuni* NCTC11168H (1 × 10^5^ CFU/bird)	No significant reduction	[239]
Ent–KLH conjugate vaccine	White Leghorn (layer)	Day 7, Day 21, and Day 35	Intramuscular administration of 100 μg of Ent–KLH conjugate vaccine with Montanide adjuvant	Day 49	*C. jejuni* (1 × 10^4^ CFU/bird)	3–4 log_10_ unit reduction in the cecum (statistically significant)	[227]
	White Leghorn (layer)	Day 7 and Day 21	Intramuscular administration of 100 μg of Ent–KLH conjugate vaccine with Montanide adjuvant	Day 35	*C. jejuni* (1 × 10^4^ CFU/bird)	3–4 log_10_ unit reduction in the cecum (statistically significant)	
Recombinant YP437 protein	Ross 308 (broiler)	Day 5 and Day 12	Intramuscular administration of 100 µg of recombinant YP437 protein (YP437 I2, P I2, YP437 I4, and P I4) emulsified with adjuvant MONTANIDETM ISA 78 VG	Day 19	*C. jejuni* (1 × 10^4^ CFU/bird)	No significant reduction	[240]
Plasmid DNA prime/recombinant protein boost vaccination (YP437 and YP9817)	Ross 308 (broiler)	Day 12	Intramuscular administration of 100 µg of recombinant protein emulsified in MONTANIDE™ ISA 78 VG	Day 19	*C. jejuni* C97Anses640 (1 × 10^4^ CFU/bird)	No significant reduction	[179]
	Ross 308 (broiler)	Day 5	Intramuscular administration of 50 μg of plasmid DNA				
*Lactococcus lactis* expressing JlpA	Vencobb (broiler)	Day 7	Oral gavage of 1 × 10^9^ CFU/100 µL of *Lactococcus lactis* expressing recombinant JlpA	Day 28	*C. jejuni* isolate BCH71 (1 × 10^8^ CFU/bird)	No significant reduction	[241]
			Subcutaneous administration of 50 µg of recombinant JlpA emulsified in incomplete Freund’s adjuvant				
Bacterin vaccine (mix of 13 *Campylobacter* suspensions)	Ross 308 (broiler)	28, 30, 32, and 34 weeks	Intramuscular administration of 8.1 log_10_ CFU inactivated *Campylobacter* (7 log_10_ CFU/*Campylobacter* strain)	Day 7 Day 14 Day 21	* C. jejuni * strain KC40 (10^2.5^ and 10^3.5^ CFU/bird)	No significant reduction	[203]
Subunit vaccine (6 immunodominant *Campylobacter* antigens)	Ross 308 (broiler)		Intramuscular administration of 75 µg of protein with Freund’s complete and incomplete adjuvant				
Diphtheria toxoid *C. jejuni* capsular polysaccharide- vaccine (CPSconj)	Ross 308 (broiler)	Day 7 and Day 21	Subcutaneous administration of 25 μg of CPSconj with 10 μg CpG or 100 μL Addavax adjuvant	Day 29	* C. jejuni * 81–176 (2 × 10^7^ CFU/bird)	0.64 log_10_ reduction (statistically significant)	[234]
Chitosan/pCAGGS-*flaA* nanoparticles	White Leghorn (layer)	Day 1, Day 15, and Day 29	Intranasal administration of 150 μg chitosan/pCAGGS-*flaA* nanoparticles	Day 42	* C. jejuni * ALM-80 (5 × 10^7^ CFU/bird)	2 log_10_ in the cecum (statistically significant)	[230]
LT-B/FlaA hybrid protein	Breed not specified (broiler)	Day 7 and Day 21	Oral administration of 250 μg, 500 μg, 750 μg, and 1 mg of LT-B/flaA *hybrid* protein; intramuscular administration of 250 µg, and 1 mg of LT-B/Fla *hybrid* protein	Day 28	*C. jejuni* A74 (2 × 10^8^ CFU/bird)	Statistically significant reduction in the number of *Campylobacter* positive birds	[228]
CjaA, CjaD, and hybrid protein rCjaAD of *C. jejuni*	Hy-line (layer)	Day 1, Day 9, and Day 19	Oral or subcutaneous administration of 2.5 × 10^9^ CFU of *L. salivarius* GEM particles with CjaALysM and CjaDLysM	Day 30	* C. jejuni * 12/2 (1 × 10^4^ CFU/bird)	No significant reduction	[229]
	Rosa 1 (broiler)	18-day-old embryo	In ovo administration of 0.1 mL of inoculum rCjaAD with GEM particles or liposomes into the amniotic fluid	Day 14	*C. jejuni* 12/2 (1 × 10^6^ CFU/bird)	Statistically significant reduction in cecal loads of *Campylobacter*	
Live attenuated *Salmonella* Typhimurium strain expressing *C. jejuni* CjaA	Cobb 500 (broiler)	Day 1 and Day 14	Oral administration of ~10^8^ CFU of *S.* Typhimurium strain χ9718 harboring pUWM1161 (Asd^+^ vector carrying the *cjaA* gene)	Day 28	*C. jejuni* Wr1 (1 × 10^5^ CFU/bird)	No significant reduction	[242]
Live attenuated *Salmonella* expressing linear peptides of *C. jejuni* (Cj0113, Cj0982c, and Cj0420)	Cobb-500 (broiler)	Day 1	Oral gavage of 10^8^ CFU/mL *Salmonella*	Day 21	* C. jejuni * PHLCJ1-J3 (2.5 × 10^6^ CFU/bird)	4.8 log reduction in the ileum with Cj0113 (statistically significant)	[161]
						4 log reduction—undetectable level in the ileum with Cj0113 (statistically significant)	
Live attenuated *Salmonella* expressing linear peptides of *C. jejuni* (Cj0113)			Oral gavage of 10^8^ CFU/mL *Salmonella* 10^8^ CFU/mL				
CmeC and CfrA	Cobb 500 (broiler)	18-day-old embryo	In ovo administration of 50 µg pCmeC-K or 50 µg pCfrA into the amniotic fluid	Day 14	*C. jejuni* NCTC 11,168 (5 × 10^7^ CFU/bird)	No significant reduction	[178]
			In ovo administration of DNA vaccines emulsified with incomplete Freund’s adjuvant	Day 21		No significant reduction	
pcDNA3-YP DNA vaccines YP_001000437.1, YP_001000562.1, YP_999817.1, and YP_999838.1	Ross PM3 (broiler)	Day 5 and Day 12	Intramuscular administration of with 300 μg of pcDNA3-YP, supplemented with 50 μg of unmethylated CpG ODN2007 followed by intramuscular administration of 100 μg of recombinant proteins emulsified in MONTANIDE™ ISA70 VG	Day 19	*C. jejuni* C97Anses640 (1 × 10^5^ CFU/bird)	2.03, 3.61, 4.27, and 2.08 log 10 reductions of P562, YP437, YP9817, and P9838 groups, respectively (statistically significant)	[231]
			Intramuscular administration of with 300 μg of pcDNA3-_999817.1, supplemented with 50 μg of unmethylated CpG ODN2007 followed by intramuscular administration of 100 μg of recombinant proteins emulsified in MONTANIDE™ ISA70 VG			No significant reduction	
CmeC	Breed not specified (broiler)	Day 7 and Day 21	Oral gavage with 50 or 200 μg of CmeC vaccine with or without with 10 μg of mLT	Day 35	*C. jejuni* NCTC 11,168 (1 × 10^6^ CFU/bird)	No significant reduction	[243]
	White Leghorn chickens (layer)	Day 21 and Day 35	Oral and subcutaneous administration of 50 or 200 μg of CmeC vaccine with or without 70 μg of mLT	Day 49	*C. jejuni* NCTC 11,168 (1 × 10^5^ CFU/bird)	No significant reduction	
* Lactococcus lactis * NZ3900/pNZ8149 expressing cjaA	White leghorn (layer)	Day 5–11 and Day 19–25	Oral administration of 2 × 10^10^ CFU of *L. lactis* NZ3900-sCjaA-Ltb, NZ3900-sCjaA, NZ3900-pNZ8149s, and NZ3900-pNZ8149	Day 33	*C. jejuni* NCTC 11,168 (1.5 × 10^6^ CFU/bird)	2.35 log_10_ and 2.05 log_10_ reduction with NZ3900-sCjaA vaccine group at post 5 DPI (statistically significant)	[163]
Glycoproteins of FlpA and SodB	White Leghorn (layer)	Day 6 and Day 16	Intramuscular administration of 240 µg of FlpA and G-FlpA or 138 µg of SodB and G-SodB.	Day 20	*C. jejuni* M1 (1 × 10^7^ CFU/bird)	No significant reduction	[244]
					*C. jejuni* M1 (10^2^ CFU/bird)	No significant reduction	
* C. jejuni * N-glycans + *Pseudomonas aeruginosa* exotoxin A (G-ExoA)	White Leghorn (layer)	Day 6 and Day 16	Intramuscular administration of 95 µg protein of ExoA or G-ExoA with MontanideTM ISA 70 VG adjuvant	Day 20	*C. jejuni* M1 (1 × 10^2^ CFU/bird)	Reduction on Day 37 with ExoA-vaccinated group (statistically significant)	[235]
					*C. jejuni* 11168H. *C. jejuni* M1 (1 × 10^4^ CFU/bird)	Reduction on Day 37 with ExoA and G-ExoA-vaccinated groups (statistically significant)	
Bacterin and subunit vaccine	Ross 308 (broiler)	18-day-old embryo	In ovo administration of 7.4 log_10_ CFU inactivated *Campylobacter*/bacterin dose of bacterin vaccine injected into the amniotic cavity	Day 19	*C. jejuni* KC4 (1 × 10^7^ CFU/bird)	No significant reduction	[245]
			In ovo administration of 28.5 μg of 6 immunodominant *Campylobacter* antigens with ESSAI IMS 1505101OVO1 adjuvant				
* C. jejuni * Dps	Cornish × Rock (broiler)	Day 10 and Day 24	Subcutaneous administration of 0.2 mg recombinant Dps protein with Freund’s complete adjuvant	Day 34	*C. jejuni* NCTC11168 (1 × 10^5^ CFU/bird)	No reduction	[224]
		Day 3, Day 10, and Day 16	Oral gavage of *Salmonella* Typhimurium strain χ9088 expressing *C. jejuni* Dps in 0.5 mL	Day 26		2.92 log_10_ reduction (statistically significant)	
PLGA-encapsulated CpG (E-CpG) ODN and *C. jejuni* lysate	Breed is not specified (layer)	Day 14	Oral administration of 5 µg or 50 µg of soluble CpG	Day 15	* C. jejuni * (10^7^ CFU/bird)	1.23 and 1.32 log reduction at 8 days post-infection with low and high doses, respectively (statistically significant)	[232]
	Breed is not specified (layer)		Oral administration of 5 µg E-CpG			0.9, 1.9, and 1.89 log reduction at 8, 15, and 22 days of post-infection (statistically significant)	
	Breed is not specified (layer)		Oral administration with a high dose of E-CpG (25 µg)			1.46 log_10_ reduction at day 22 post-infection (statistically significant)	
	Breed is not specified (broiler)		Oral administration of a low dose of *C. jejuni* lysate (4.3 µg protein)			2.14 and 2.14 log_10_ at day 8 and day 22 post-infection, respectively (statistically significant)	
	Breed is not specified (broiler)		Oral administration of E-CpG ODN (25 µg) and *C. jejuni* lysate (4.3 µg protein)			2.42 log_10_ at day 22 post-infection (statistically significant)	
*C. jejuni* Type VI secretion system (T6SS) protein Hcp encapsulated nanoparticles	Vencobb (broiler)	Day 7, Day 14, and Day 21	Oral gavage of 50 μg rHcp loaded CS-TPP NPs (CS-TPP-Hcp)	Day 28	* C. jejuni * isolate BCH71 (1 × 10^8^ CFU/bird)	1 log reduction (statistically significant)	[233]
			Subcutaneous administration of 50 μg of rHcp emulsified with incomplete Freund’s adjuvant			0.5 log reduction (statistically significant)	
Recombinant NHC flagellin	Ross 308 (broiler)	18.5-day-old embryo	In ovo administration of 40 or 20 μg NHC flagellar protein with 10 mM Tris (pH 9.0), 20% glycerol, 5 mM sucrose	day 18	*C. jejuni* (1 × 10^5^ CFU/bird)	No significant reduction	[246]
Recombinant *C. jejuni* peptides of CadF, FlaA, FlpA, CmeC, and CadF-FlaA-FlpA fusion protein	Cornish cross (broiler)	Day 6 and Day 16	Intramuscular administration of 240 µg of GST-tagged 90-mer peptide or equal mixture of CadF-His, FlaA-His, and FlpA-His (trifecta group) emulsified in Montanide ISA 70 VG	Day 20	* C. jejuni * (2 × 10^8^ CFU/bird)	3.1, 3.3, 3.1, and 1.7 log reductions observed with Trifecta, FlpA, FlaA and CadF, respectively (statistically significant)	[157]

CfrA: Ferric enterobactin receptor; CjaA: *C. jejuni* amino acid-binding protein; CjaD: Peptidoglycan-binding protein; CmeC: An essential component of CmeABC multidrug efflux pump; CpG ODN: Oligodeoxynucleotides containing unmethylated CpG motifs; CS-TPP NPs: Chitosan–Sodium tripolyphosphate nanoparticles; DPI: Days post-infection; Dps: DNA-binding protein; Ent–KLH conjugate vaccine: Enterobactin conjugated to the carrier keyhole limpet hemocyanin; FlaA: Flagellin A; FliD: Flagellum-capping protein; FlpA-10 × GT: FlpA with 10 N-Heptasaccharide Glycan Moieties; FspA: Flagellum-secreted protein; GEM particles: Gram-positive Enhancer Matrix particles; JlpA: *C. jejuni* lipoprotein A; LT-B: Binding subunit of the heat-labile enterotoxin; mLT: Modified *E. coli* heat-labile enterotoxin; ODN: Oligodeoxynucleotides; OMPs: Outer-membrane proteins; SodB: Superoxide dismutase.

## 4. Conclusions and Future Perspectives of Campylobacter Control

As a food-borne pathogen, *Campylobacter* continues to pose a challenge to global public health, with poultry serving as the primary source of human infections. Growing concerns regarding antimicrobial resistance and the push for antibiotic-free poultry production have accelerated the need for sustainable and long-term control measures against *Campylobacter* in poultry. This comprehensive review focuses on possible preharvest options to control *Campylobacter* colonization in chickens, with a special emphasis on vaccination. As a single strategy cannot completely prevent *Campylobacter* colonization, our review highlights the importance of a multifaceted approach that integrates several on-farm interventions. Strict biosecurity measures play a fundamental role in preventing the introduction and spread of *Campylobacter*. Additionally, dietary interventions such as probiotics, prebiotics, postbiotics, and feed additives offer promising avenues for modulating the gut microbiome and enhancing host resistance to *Campylobacter* colonization. Importantly, vaccination stands out as one of the most logical approaches for preventing and reducing *Campylobacter* colonization at the source level. Although there is currently no commercial vaccine available, ongoing research on multi-epitope and universal vaccine designs, coupled with advancements in delivery systems and formulations, offers great promise in addressing the challenges presented by the genetic diversity of the pathogen and unique immunological characteristics of poultry.

### 4.1. Future Prospects

#### 4.1.1. Biosecurity Enhancing Innovations

Biosecurity innovations provide a more efficient primary protective barrier against the entry of *Campylobacter* into poultry farms [247]. Improved fly control management through biological traps and insecticide-impregnated netting has significantly reduced the prevalence of *Campylobacter* in farms. Furthermore, managing the poultry house environment using new technologies such as electrostatic air filtration, UV-based disinfection, automated cleaning systems, and water purification systems offers promising tools for reducing environmental exposure to *Campylobacter*. More advanced features, such as real time monitoring systems for detecting contamination hotspots on farms enable early action against *Campylobacter* and prevent its entry and spread [248]. However, effective implementation depends on human compliance, including proper training and stringent adherence to biosecurity protocols by farm workers [249,250].

#### 4.1.2. Studies Targeting *Campylobacter* and Host Interactions

Limited knowledge of *Campylobacter* pathophysiology and host immune responses has been a major obstacle to the development of effective control strategies [251]. However, recent advances have significantly deepened our understanding of the mechanisms governing colonization and host–pathogen interactions of these pathogens. Transcriptomic and immunogenomic investigations in poultry have identified intestinal immune gene expression signatures linked to reduced colonization and detailed *Campylobacter*-induced cytokine responses in avian cells [252,253]. Comparative analyses of innate immune activation have further revealed unique early transcriptional patterns that distinguish *Campylobacter* from other enteric pathogens, such as *Salmonella* [254].

In addition,, multi-omics approaches have begun to clarify the molecular basis of *Campylobacter* adaptation and virulence. Integrated analyses of the cecal microbiota and host responses have connected specific microbial communities and metabolic pathways to bacterial growth dynamics and colonization in chickens [255]. Proteomic and metabolomic investigations have demonstrated that *C. jejuni* undergoes extensive molecular remodeling in chicken exudates, reflecting adaptations that enhance survival and virulence [256]. Moreover, host serum multi-omics profiling has highlighted the modulation of immune and metabolic pathways during colonization and therapeutic interventions [257]. At the systems level, integrative analyses have also identified potential core targets for vaccine development [258]. Collectively, these studies provide a more detailed perspective on avian immunity and *Campylobacter*–host interactions, informing the rational design of future mitigation strategies and next-generation vaccines.

#### 4.1.3. Genetic Selection of Campylobacter-Resistant Breeds

A long-term approach to control *Campylobacter* involves the genetic selection of breeds resistant to bacterial colonization. Research has demonstrated that Quantitative Trait Loci (QTL), major histocompatibility complex (MHC), and immune response genes vary among birds with various levels of resistance to *Campylobacter* [259,260]. The selection of breeder stocks resistant to *Campylobacter* can help to control colonization at the primary production level.

#### 4.1.4. Developing Effective Vaccination Strategies

One of the main challenges in developing an effective *Campylobacter* vaccine is the high antigenic diversity among strains, which hinders cross-protection. This issue can be addressed by identifying conserved and protective antigens shared between multiple strains [261]. Further research is needed to identify broad-spectrum vaccine targets (e.g., multi-epitope vaccines) using in silico prediction tools. Reverse vaccine technology also offers avenues for identifying vaccine antigen candidates that offer protection against a wide range of *Campylobacter* strains [260,262]. Additionally, optimizing mucosal vaccine delivery systems can enhance vaccine efficacy against *Campylobacter* colonization [233,263].

#### 4.1.5. Microbiota Targeting Interventions

A healthy gut microbiota can inhibit *Campylobacter* colonization through competitive exclusion and the production of antimicrobial metabolites (e.g., short-chain fatty acids), thereby improving mucosal immunity. These beneficial effects can be achieved through the use of prebiotics, probiotics, and postbiotics, which help modulate gut microbiota and support protective microbial communities [264,265]. Emerging technologies like fecal microbiota transplantation (FMT) and precision microbiome engineering are still in the early stages, but represent promising future avenues for *Campylobacter* control [114,266].

#### 4.1.6. Cross-Sectoral Collaboratory Efforts (One Health)

Effective preharvest control strategies require strong and sustained collaboration among researchers, the poultry industry, and policymakers. Success depends on teamwork, advanced planning, and a combination of efforts across all three sectors. Future control depends on teamwork, proactive planning, and coordinated efforts across all three sectors. The adoption of a One Health approach, combined with the practical application of scientific innovations at the farm level, can greatly reduce the global burden of *Campylobacter* [13,251,267].

## Data Availability

No new data were created or analyzed in this study. Data sharing is not applicable to this article.

## Abbreviations

The following abbreviations are used in this manuscript:

AMR	Antimicrobial resistance
CFU	Colony forming units
EOs	Essential oils
FMT	Fecal microbiota transplantation
FOS	Fructooligosaccharides
GBS	Guillain-Barré Syndrome
GIT	Gastrointestinal tract
GLAT	Gut-associated lymphoid tissue
GOS	Galactooligosaccharides
IBS	Irritable bowel syndrome
IMO	Isomalto-oligosaccharides
MHC	Major histocompatibility complex
PPE	Personal protective equipment
QTL	Quantitative Trait Loci
VBNC	Viable but non-culturable state
